# Can the results of quantum refinement be improved with a continuum-solvation model?

**DOI:** 10.1107/S2052520621009574

**Published:** 2021-11-17

**Authors:** Justin Bergmann, Esko Oksanen, Ulf Ryde

**Affiliations:** a Department of Theoretical Chemistry, Chemical Center, Lund University, PO Box 124, S-22100 Lund, Sweden; b Instruments Division, European Spallation Source ESS ERIC, PO Box 176, SE-221 00 Lund, Sweden

**Keywords:** quantum refinement, continuum solvation, nitrogenase, particulate methane monooxygenase, acetylcholin esterase, quantum crystallography

## Abstract

Quantum refinement has repeatedly been shown to be a powerful approach to interpret and improve macromolecular crystal structures, allowing for the discrimination between different interpretations of the structure, regarding the protonation states or the nature of bound ligands, for example. In this method, the empirical restraints, used to supplement the crystallographic raw data in standard crystallographic refinement, are replaced by more accurate quantum mechanical (QM) calculations for a small, but interesting, part of the structure. Previous studies have shown that the results of quantum refinement can be improved if the charge of the QM system is reduced by adding neutralizing groups. However, this significantly increases the computation time for the refinement. In this study, we show that a similar improvement can be obtained if the original highly charged QM system is instead immersed in a continuum solvent in the QM calculations. The best results are typically obtained with a high dielectric constant (ɛ). The continuum solvent improves real-space *Z* values, electron-density difference maps and strain energies, and it normally does not affect the discriminatory power of the calculations between different chemical interpretations of the structure. However, for structures with a low charge in the QM system or with a low crystallographic resolution (>2 Å), no improvement of the structures is seen.

## Abstract 2

Quantum refinement has been shown to be a powerful approach to interpret and improve macromolecular crystal structures. Previous studies have shown that the results of quantum refinement can be improved if the charge of the quantum mechanical (QM) system is reduced by adding neutralizing groups. Here it is shown that a similar improvement can be obtained if the original highly charged QM system is instead immersed in a continuum solvent in the QM calculations.

## 1. Introduction

X-ray crystallography is the prime method for obtaining three-dimensional structures of proteins. Owing to the limited resolution of protein crystal structures, it is normally necessary to introduce empirical restraints in the crystallographic refinement to ensure that the structures make chemical sense (*i.e.* give reasonable bond lengths and angles) (Kleywegt & Jones, 1997 ▸). These restraints are usually derived from high-resolution structures (Engh & Huber, 1991 ▸). In the language of computational chemistry, they represent a molecular mechanics force field. Therefore, standard crystallographic refinement optimizes an energy function of the form: 

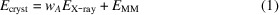

where *E*_X-ray_ is a crystallographic goodness-of-fit criterion, reflecting how well the current model (coordinates, occupancies and atomic displacement parameters), reproduce the experimental data, *E*_MM_ is the empirical restraints and *w*_A_ is a weight factor determining the relative importance of the two terms. This shows that protein crystal structures to a significant degree are actually computational and that they depend on the accuracy of the empirical restraints.

The empirical restraints are accurate for protein residues and nucleic acids, for which there are ample accurate experimental data. However, for cofactors, substrates and inhibitors, much less information is available, making the restraints less certain. Even worse, for metal sites it is hard to construct an empirical potential (Hu & Ryde, 2011 ▸) and it depends strongly on all the ligands and the charge and spin state of the metal. Therefore, these parts of the crystal structures have a lower accuracy than the amino acid parts.

To overcome these problems, we have suggested that the empirical restraints can be replaced by quantum-mechanical (QM) calculations (Ryde *et al.*, 2002 ▸). This can be done for a small, but interesting, part of the structure (*e.g.* the active site) in the same way as in standard combined quantum-mechanical and molecular-mechanical (QM/MM) methods (Senn & Thiel, 2009 ▸; Ryde, 2016 ▸). This part will be called system 1 (or the QM system) in the following. This leads to the quantum-refinement energy function: 

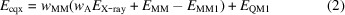

Here, *E*_QM1_ is the QM energy of system 1 and we need to subtract the corresponding MM energy of system 1, *E*_MM1_, to avoid double counting of energy terms. Finally, *w*_MM_ is another weight factor that is necessary because the empirical restraints are normally in statistical units, whereas the QM energy is in energy units.

Another problem with standard X-ray crystallography is that it normally cannot discern H atoms and that it is not possible to distinguish between isoelectronic groups. These problems apply in principle also to quantum refinement. However, the addition of protons or replacement of one group with an isoelectronic group leads to subtle changes in the surrounding structure, owing to changes in the net charge, the hydrogen-bond pattern or changes in the chemistry. By the use of QM calculations, accurate information regarding the chemical preferences is introduced into the refinement (such information could in principle also be incorporated into the empirical restraints, but it is seldom available or accurate enough, especially as the restraints normally exclude electrostatics, which provide the most sensitive information about the chemical structure). With quantum refinement, different structural interpretations can be compared to decide which fits the crystal structure best using standard crystallography quality measures, like electron-density difference maps and real-space *Z* scores (Tickle, 2012 ▸), and QM measures, like strain energy (Ryde *et al.*, 2002 ▸; Ryde, 2002 ▸; Borbulevych *et al.*, 2016 ▸). We have shown that quantum refinement can locally improve crystal structures (Ryde & Nilsson, 2003 ▸) and that it can distinguish the protonation state of metal-bound ligands (Nilsson & Ryde, 2004 ▸; Cao *et al.*, 2017 ▸, 2018*b* ▸; Caldararu *et al.*, 2018 ▸), the oxidation state of metal sites (Rulíšek & Ryde, 2006 ▸; Cao *et al.*, 2019 ▸), detect photoreduction of metal ions (Nilsson & Ryde, 2004 ▸; Söderhjelm & Ryde, 2006 ▸; Rulíšek & Ryde, 2006 ▸) and solve scientific problems regarding what is really seen in crystal structures (Söderhjelm & Ryde, 2006 ▸; Cao *et al.*, 2019 ▸). Several other groups have implemented this and similar approaches (Yu *et al.*, 2005 ▸; Borbulevych *et al.*, 2014 ▸; Hsiao *et al.*, 2010 ▸; Zheng *et al.*, 2017 ▸; Genoni *et al.*, 2018 ▸; Fadel *et al.*, 2015 ▸) and shown that it can be used to reduce errors in crystal structures and decide protonation and tautomeric states (Yu *et al.*, 2006 ▸; Borbulevych *et al.*, 2016 ▸, 2021 ▸).

Other approaches have also been developed to improve crystal structures with QM data (Genoni *et al.*, 2018). In particular, QM calculations can be used to improve the calculated electron density of the model. In standard crystallography, it is assumed that the density is spherical around each atom, the independent-atom model (IAM) (Cromer, 1965 ▸). This is a poor approximation for H atoms, for which the density is displaced towards the bond partner, leading to bond lengths that are ∼0.1 Å too short. However, it can also be improved for other atoms in accurate crystal structures, giving information about chemical bonding and lone pairs. This can be done by using a multipole model (Hansen & Coppens, 1978 ▸), but it requires too many refined parameters for biomacromolecules. Therefore, various databases of multipolar parameters have been constructed from QM calculations or experimental structures (Jarzembska & Dominiak, 2012 ▸; Domagała *et al.*, 2012 ▸; Dittrich *et al.*, 2013 ▸). Even more accurate results can be obtained if the QM density is employed directly in the refinement, which is the aim of Hirshfeld atom refinement (HAR) (Jayatilaka & Dittrich, 2008 ▸; Capelli *et al.*, 2014 ▸). This approach is also too demanding to apply to biomacromolecules, but a fragmentation approach has been developed (Bergmann *et al.*, 2020 ▸) and a combination of HAR with a library of extremely localized molecular orbitals have been applied to small proteins like crambin (Malaspina *et al.*, 2019 ▸).

We have recently observed that the results of quantum refinement can be improved if a QM system with a large net change is partially neutralized by including nearby residues with the opposite charge (Cao *et al.*, 2020 ▸). Naturally, the time consumption of quantum-refinement calculations strongly depend on the size of the QM system. Therefore, it not optimal to add residues to the QM system only to compensate the charge. An alternative may be to include a continuum-solvent model. In this study, we test whether the results of quantum refinement can be improved by employing a COSMO continuum solvent in the QM calculations (Klamt & Schüürmann, 1993 ▸). We tested it for five crystal structures of Mo and V nitrogenase, particulate methane monooxygenase and acetylcholin esterase, *i.e.* systems with or without metals and with varying net charges. We investigate how the results depend on the dielectric constant employed for the solvent model and how the discriminating power of the quantum refinement is affected.

## 2. Methods

### 2.1. Quantum refinement

The quantum-refinement calculations were run with the *ComQum-X* software (Ryde *et al.*, 2002 ▸), which is an interface between the QM software *Turbomole* (Furche *et al.*, 2014 ▸) and the crystallography and NMR system (*CNS*) software (Brünger *et al.*, 1998 ▸; Brunger, 2007 ▸). For V nitrogenase with an OH^−^ or NH^2−^ ligand, there are two conformations in the active site (Cao *et al.*, 2020 ▸) and we therefore used the *ComQumX-2QM* approach (Cao & Ryde, 2020 ▸). The full protein was considered in all calculations, including all crystal water molecules. For the protein, we used the standard *CNS* force field (protein_rep.param, water_rep.param and ion.param). The empirical restraints for nonstandard residues were downloaded from the hetero compound information centre Uppsala (Kleywegt, 2007 ▸). For the *w*_*A*_ factor (determining the relative weight between the crystallographic data and the empirical potential), we used the default value suggested by *CNS* (specified below). The *w*_MM_ weight was set to 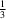 as in all our previous studies (Ryde *et al.*, 2002 ▸; Cao *et al.*, 2017 ▸). For the crystallographic target function, we used the standard maximum-likelihood function using amplitudes (mlf) in *CNS* (Pannu & Read, 1996 ▸; Adams *et al.*, 1997 ▸). *CNS* does not support anisotropic atomic displacement parameters (ADPs), so only isotropic ADPs were used. After quantum refinement, ADP refinement was performed using phenix.refine (Afonine *et al.*, 2012 ▸). The electron-density maps were generated using phenix.maps.

### 2.2. Protein setup

**Figure 1:**
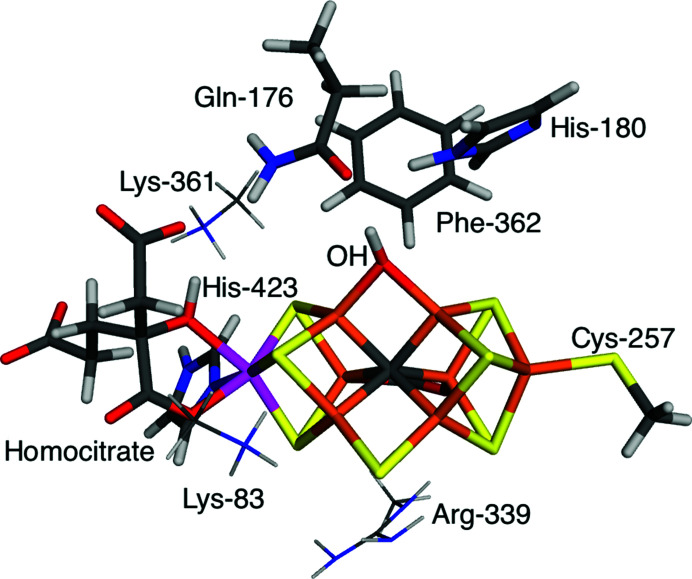
The QM systems used for V nitrogenase. The small QM model contained only atoms shown with thick sticks, whereas the large QM model included also the three residues shown as thin sticks.

The quantum-refinement calculations of V nitrogenase with a putative N-derived reaction intermediate were based on the 6fea crystal structure at 1.2 Å resolution (Sippel *et al.*, 2018 ▸). The FeV cluster was modelled by VFe_7_S_7_C(CO_3_)(homo­citrate)(CH_3_S)(imidazole), where the two last groups are models of Cys-257 and His-423 (all residues are from the D subunit of the crystal structure). In addition, the putative N-derived ligand, as well as models of Gln-176, His-180 and Phe-362, were included in the QM calculations, as can be seen in Fig. 1 ▸, a total of 90 atoms. For the larger QM system, we also included the nearby Lys-83, Arg-339 and Lys-361 residues [modelled by CH_3_NH_3_^+^ or CH_3_NHC(NH_2_)_2_^+^; shown as thin sticks in Fig. 1 ▸]. Two different interpretations of the bound ligand were tested: OH^−^ or NH^2−^. OH^−^ was assumed to bind to the E_0_ resting state with a formal oxidation state of V^III^Fe^II^_4_Fe^III^_3_ (Benediktsson *et al.*, 2018 ▸). For NH^2−^, a two-electron oxidized state was assumed, corresponding to the E_6_ state (Cao *et al.*, 2020 ▸). We have shown that the crystal structure contains a significant amount (14%) of the undissociated S2B ligand, which was included in all refinements using a separate QM calculation, modelled in the resting E_0_ state, and Gln-176 in the nonrotated conformation, observed in the crystal structure of the resting state (Sippel & Einsle, 2017 ▸). We assumed a quartet spin state for the FeV cluster (Benediktsson *et al.*, 2018 ▸; Cao *et al.*, 2020 ▸). The *w*_*A*_ factor was 0.0794.

The quantum-refinement calculations for the resting state of V nitrogenase were based on the 5n6y crystal structure, obtained at a resolution of 1.35 Å (Sippel & Einsle, 2017 ▸). We used two sizes of the QM system also for these calculations. The small QM system included only the FeV cluster (from the A subunit of the protein; VFe_7_S_8_C), the bidentate ligand, homocitrate, as well as the side chain of Cys-257 (CH_3_S^−^) and the imidazole ring of His-423. In the large QM system, we included also the side chains of Lys-83 and Lys-362 (modelled as CH_3_NH_3_^+^), as well as the whole Arg-339 (except O, but including a –COCH_3_ group from Pro-338) and the side chain of Thr-335. The positively charged Lys and Arg residues were included to compensate the high negative charge of the FeV cluster, whereas Thr-335 and the backbone of Arg-339 form hydrogen bonds to the bidentate ligand. We tested three different interpretations of the bidentate ligand: CO_3_^2−^, HCO_3_^−^ and NO_3_^−^. In all cases, we employed the oxidation-state assignment V^III^Fe^II^_4_Fe^III^_3_, corresponding to the E_0_ resting state, and we assumed a quartet spin state for the FeV cluster (Benediktsson *et al.*, 2018 ▸; Cao *et al.*, 2020 ▸). The *w*_*A*_ factor was 0.145.

The calculations of Mo nitrogenase were based on the 3u7q crystal structure at 1.00 Å resolution (Spatzal *et al.*, 2011 ▸). We studied the FeMo cluster in the C subunit of the protein. The QM system was the FeMo cluster (MoFe_7_S_8_C), homocitrate, the imidazole ring from His-442 and the side chain of Cys-275. In the large QM system, we included also the side chains of Arg-96 and Arg-359 [modelled as CH_3_NHC(NH_2_)_2_^+^]. Two protonation states were tested for the homocitrate ligand. One (1Ha) had a proton on the alcohol oxygen (O7), shared with a carboxylate oxygen (O1). The other (2H) had an additional proton on the O2 carboxylate atom (Cao *et al.*, 2017 ▸). We assumed the standard formal oxidation-state assignment for the E_0_ resting state, Mo^III^Fe^II^_3_Fe^III^_4_ (Bjornsson *et al.*, 2014 ▸, 2017 ▸) and a quartet electronic state (Hoffman *et al.*, 2014 ▸). The *w*_*A*_ factor was 0.0793. The 1Ha protonation state was used for the homocitrate ligand in the quantum refinement calculations of V nitrogenase (Benediktsson & Bjornsson, 2020 ▸; Cao *et al.*, 2020 ▸).

The calculations on particulate methane monooxygenase (pMMO) were based on the 3rgb crystal structure at a resolution of 2.8 Å (Smith *et al.*, 2011 ▸). The QM system involved one or two Cu ions and His-33, His-137 and His139 from the E subunit of the structure (only the imidazole rings of the latter two, but NH_2_CH_2_CH_2_–imidazole for His-33, *i.e.* including the amino terminal group, which also coordinates to Cu). The structures were taken from our previous investigation (Cao *et al.*, 2018*a* ▸). The copper ions were studied in the closed-shell reduced state and the *w*_*A*_ factor was 4.91.

The calculations on acetylcholine esterase were based on the 5fpq crystal structure at a resolution of 2.4 Å (Allgardsson *et al.*, 2016 ▸). The QM system consisted of all atoms of residues Glu-202 and Ser-203 {the latter with a covalently attached sarin molecule, –OP(O)(CH_3_)[OCH(CH_3_)_2_]}, the side chains of Gln-228, Ser-229, Glu-334, His-447 and Glu-450, the backbone of Tyr-119, Gly-120, Gly-121, Gly-122, Val-330, Val-331, Gly-448 and Tyr-449, as well as three water molecules (2032, 2034 and 2060; a total of 167 atoms), all from the B subunit of the crystal structure; the *w*_*A*_ factor was 1.21.

For all five crystal structures, coordinates, occupancies, ADPs and structure-factor amplitudes were obtained from the Protein Data Bank (PDB), together with the space group, unit-cell parameters, resolution limits, *R* factors and the test set used for the evaluation of the *R*_free_ factor.

The quality of the models was judged by the real-space difference-density Z score (RSZD), calculated by *EDSTATS* (part of the CCP4 package; Winn *et al.*, 2011 ▸), which measures the local accuracy of the model (Tickle, 2012 ▸). The maximum of the absolute negative and positive RSZD value was calculated for all atoms in the QM systems (atoms outside the QM system were kept frozen according to the original crystal structure). RSZD is typically less than 3.0 in absolute terms for a good model.

### 2.3. QM calculations

All QM calculations were performed at the TPSS/def2-SV(P) level of theory (Tao *et al.*, 2003 ▸; Weigend & Ahlrichs, 2005 ▸). The speed of the calculations was increased by expanding the Coulomb interactions in an auxiliary basis set, *i.e.* the resolution-of-identity (RI) approximation (Eichkorn *et al.*, 1995 ▸, 1997 ▸). Empirical dispersion corrections were included with the DFT-D3 approach (Grimme *et al.*, 2010 ▸) and Becke–Johnson damping (Grimme *et al.*, 2011 ▸).

The continuum-solvent calculations were performed with the conductor-like screening model (COSMO) (Klamt & Schüürmann, 1993 ▸) implemented in *Turbomole*. The default-optimized COSMO radii were employed and a water solvent radius of 1.3 Å (Klamt *et al.*, 1998 ▸), whereas a radius of 2.0 Å was used for the metals (Sigfridsson & Ryde, 1998 ▸). We tested two different dielectric constants, 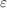 = 4 (a protein-like environment) and 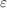 = 80 (water).

For the nitrogenase models, the QM calculations were performed with the broken-symmetry (BS) approach (Lovell *et al.*, 2001 ▸): each of the seven Fe ions was modelled in the high-spin state, with either a surplus of α (four Fe ions) or β (three Fe ions) spin. We employed the broken-symmetry BS7-235 state with a surplus of β spin on Fe2, Fe3 and Fe5 for all calculations. This is the best BS state for the resting state of Mo nitrogenase and also for several other states (Lovell *et al.*, 2001 ▸; Cao & Ryde, 2018 ▸; Cao *et al.*, 2018*b* ▸), and this state was also used in previous studies on V nitrogenase (Benediktsson & Bjornsson, 2020 ▸; Cao *et al.*, 2020 ▸; Bergmann *et al.*, 2021 ▸). This state was obtained using the fragment approach by Szilagyi & Winslow (2006 ▸) or by swapping the coordinates of the Fe ions (Greco *et al.*, 2011 ▸).

## 3. Results and discussion

In this study, we investigate whether the results of quantum refinement can be improved by carrying out the QM calculations in a COSMO continuum solvent (Klamt & Schüürmann, 1993 ▸) and whether the solvent may allow calculations with a smaller but more highly charged QM system. We test the approach for five different crystal structures to investigate when it is applicable, what is a proper choice of the dielectric constant and whether it affects the discriminatory power of quantum refinement. The results for each crystal structure are described in separate sections.

### 3.1. OH^−^ or NH^2−^ binding to V nitrogenase

We first study the 6fea crystal structure of V nitrogenase (1.2 Å resolution), showing a small ligand binding to the FeV cluster (Sippel *et al.*, 2018 ▸). This ligand was originally interpreted as a reaction intermediate, NH^2−^ or NH_2_^−^. However, both QM/MM and quantum-refinement studies showed that it is rather OH^−^ (Benediktsson *et al.*, 2018 ▸; Cao *et al.*, 2020 ▸). The ligand replaces one of the μ_2_-bridging sulfide ions, S2B, but we showed that it contains a significant amount of undissociated S2B (∼14%). Therefore, the quantum-refinement calculations were performed with the *ComQumX-2QM* approach (Cao & Ryde, 2020 ▸), performing two separate QM calculations for the two alternative conformations. However, most importantly for this study, we showed that the structure was improved significantly if three positively charged residues were included in the QM system, to compensate the large negative charge of the QM system.

Here we investigate whether we can get a similar improvement with the original QM system, but using a COSMO model instead. Thus, we compare the results obtained using either a small QM model with only the directly coordinated ligand and three residues around the ligand or a larger model in which three neutralizing groups are added to the QM system, as can be seen in Fig. 1 ▸. The net charge of the small QM system is −5 or −6, depending on the interpretation of the unknown ligand, whereas it is −2 or −3 for the large QM system. We compare results obtained with three different dielectric constants: 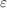 = 1 (*i.e.* no COSMO model), 4 (a protein-like environment) and 80 (a water-like environment).

**Figure 2:**
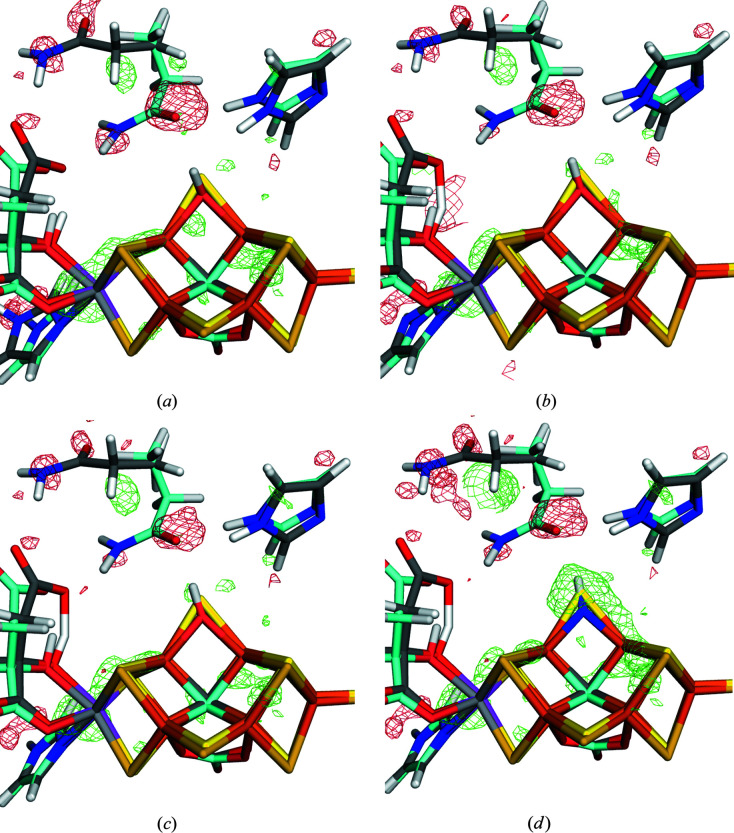
Quantum-refined structures of the FeV cluster with different sizes of the QM system and different interpretations of the bound ligand, replacing S2B: (*a*) OH^−^ with the small QM system in a vacuum, (*b*) OH^−^ with the large QM system in a vacuum, (*c*) OH^−^ with the small QM system and 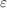 = 80, and (*d*) NH^2−^ with the small QM system and 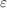 = 80. The *mF*_o_ −*DF*_c_ difference maps are contoured at +3σ (green) and −3σ (red). All systems were refined with *ComQumX-2QM* and two conformations of the QM system, namely one (86%, cyan) with the unknown ligand and the other (14%, grey) with S2B still bound.

The results are collected in Table 1 ▸. We first discuss the results obtained with the preferred OH^−^ ligand (upper half of the table). It can be seen that in a vacuum, 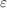 = 1, the large model (with one Arg and two Lys residues) gives appreciably lower RSZD scores than the smaller QM system for all residues, except S2B. Therefore, the sum of the RSZD scores is reduced from 18.3 to 12.7. This can also be seen in the electron-density difference maps in Figs. 2 ▸(*a*) and 2 ▸(*b*). However, a similar improvement is found also when the COSMO model is used for the small system: the sum of the RSZD scores is reduced from 18.3 in a vacuum to 11.2 with 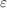 = 80. The effect comes from all residues, except S2B, but it is especially large for the two conformations of Gln-176 (Fig. 2 ▸ *c*). In fact, the result with 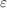 = 80 is better than that obtained with the large model in a vacuum.

It can also be seen that the strain energies (*i.e.* the difference in QM energy of the QM system between structures optimized with or without the crystallographic data) of the two conformations are reduced, from 124 to 46 kJ mol^−1^ for the conformation with OH^−^ (AC1) and from 10 to 8 kJ mol^−1^ for the S2B-bound conformation (AC2). The strain energies are always much larger for the large QM system than for the small one. This reflects that the strain energies strongly depend on the size of the QM system (in a larger QM system, there are more atoms that can be strained) (Ryde, 2002 ▸). Therefore, strain energies obtained with different sizes of QM systems are not comparable.

The large QM system is also improved with the continuum-solvation model, but to a smaller extent: the sum of the RSZD scores decrease from 12.7 to 11.1, whereas the strain energies decrease from 208 to 64 kJ mol^−1^ for the OH-bound conformation and from 20 to 10 kJ mol^−1^ for the S2B-bound con­formation.

Next, we checked that the discriminatory power was not compromised by the COSMO model, by including calculations also with the second best interpretation of the ligand, NH^2−^ (Cao *et al.*, 2020 ▸). The results in the second part of Table 1 ▸ show that this ligand gives similar trends to the OH^−^ ligand: both the RSZD scores and the strain energies decrease when going from a vacuum to 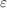 = 80, both for the small and the large QM systems. However, most importantly, it can be seen that the RSZD score of the unknown ligand is always higher for the NH^2−^ than for the OH^−^ models, by 1–4. Likewise, the sum of the RSZD scores is always also larger for models with NH^2−^ than for the corresponding OH^−^ models, by 5–10. This can also be seen from the difference maps in Figs. 2 ▸(*c*) and 2(*d*). The same applies for the strain energy for AC1, which is lower for OH^−^ than for NH^2−^ by 2–24 kJ mol^−1^, except for the small model with 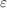 = 80. In fact, the difference decreases with increasing 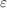 , reflecting that the strain energy decreases with increasing 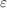 for both ligands and both sizes of the QM system. The strain energy of AC2 is nearly the same for the two models (within 1 kJ mol^−1^), reflecting that AC2 is the same for the two models.

**Figure 3:**
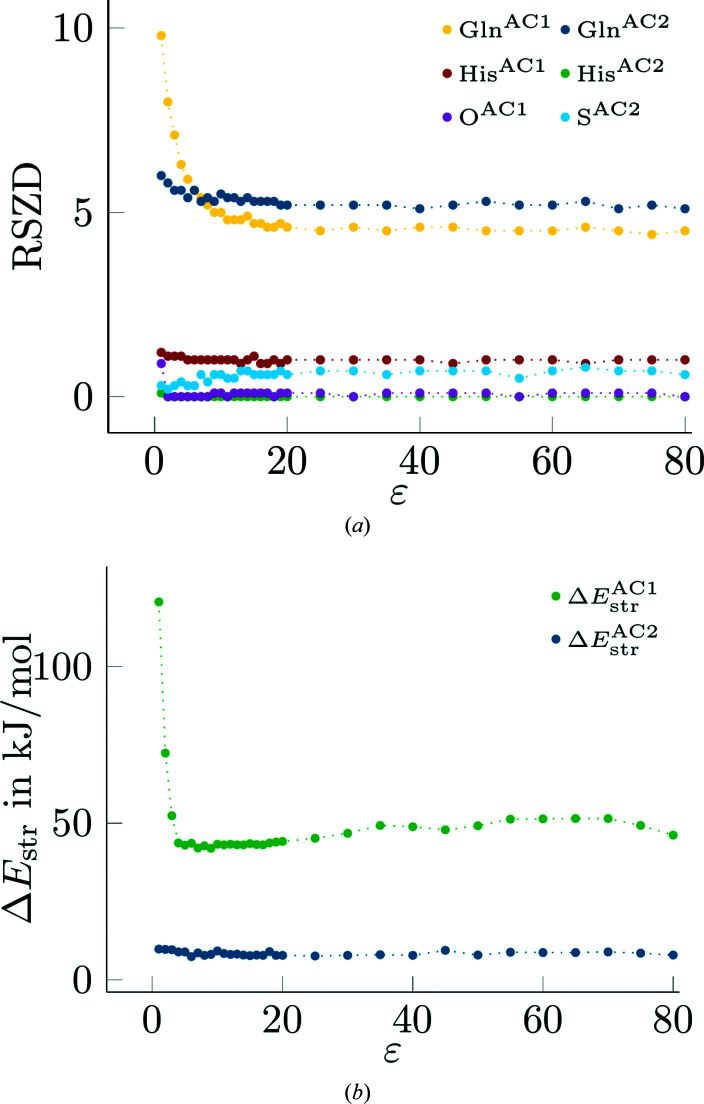
RSZD values (*a*) and strain energies (*b*) as a function of the dielectric constant ( 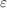 ) for quantum refinement calculations of the small-QM OH^−^ model with varying from 1 to 80 in steps of 1 and 5 in the intervals 1–20 and 20–80, respectively.

Finally, we performed a more detailed study of how the RSZD results depend on the dielectric constant by performing quantum-refinement calculations for the small-QM OH^−^ model with 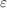 varying from 1 to 80 in steps of 1 and 5 in the intervals 1–20 and 20–80, respectively. The results are shown in Fig. 3 ▸(*a*).

It can be seen that the RSZD values for the two alternative conformations of His-180 and for OH^−^ decrease somewhat from 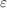 = 1 to 2 or 4 and are then essentially constant, with fluctuations of 0–0.1 (note that *EDSTATS* reports the RSZD value with a single decimal). Gln-176 in AC1 shows the largest variation, with a large decrease in RSZD from 9.8 to 4.4. The decrease is monotonous until 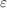 = 13 and the lowest value is not attained until 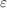 = 75, although the variation is only 0.2 for 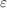 > 19. The RSZD value of the same residue in AC2 also decreases with 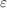 , but only from 6.0 to 5.1, and the fluctuations are 0.2 for 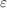 > 14. In contrast, the RSZD value of S2B (of AC2) shows a slight increase with increasing 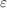 , from 0.3 to ∼0.8, but the fluctuations are 0.3 even for 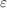 = 55–80. As a consequence, the sum of the six RSZD values decreases with increasing 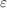 , from 18.3 to 11.2. Thus, it seems clear that 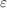 = 4 is too small to give converged results, but essentially any value >20 can be used. The random fluctuations of 0.1–0.3 for the individual RSZD values seem to be caused not by variations in the geometries, but mainly by variations in the *B* factors obtained by *Phenix* after the rerefinement of the coordinates of the QM system.

The corresponding strain energies are shown in Fig. 3 ▸(*b*). It can be seen that the strain energy of AC2 decreases slightly with increasing 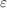 , from 10 to 8 kJ mol^−1^. However, for 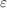 > 5, it is stabilized, with fluctuations of < 2 kJ mol^−1^. The strain energy of AC1 also first decreases with increasing 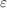 , from 124 to 42 kJ mol^−1^. However, from 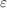 = 10, it increases again, reaching 51 kJ mol^−1^ at 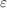 = 55–70, after which it decreases again to 46 kJ mol^−1^ at 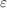 = 80. The fluctuations are similar to those of AC2, *i.e.* <2 kJ mol^−1^.

Consequently, we can conclude that a COSMO model clearly improves the result of quantum refinement for systems with a large negative charge and can therefore be used to avoid the need of enlarging the QM system with neutralizing residues.

### 3.2. The bidentate ligand in V nitrogenase

**Figure 4:**
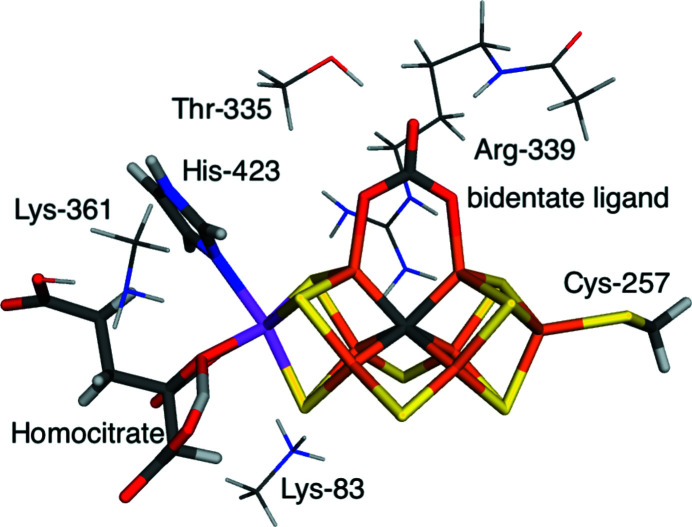
The QM systems used for the FeV cluster with different interpretations of the bidentate ligand. The small QM model contained only atoms shown with thick sticks, whereas the large QM model included also the four residues shown as thin sticks.

In the second application, we also consider V nitrogenase, but concentrate instead on the nature of the bidentate ligand that bridges two of the Fe ions in the FeV cluster. From the original crystallographic data (5n6y at 1.35 Å resolution; Sippel & Einsle, 2017 ▸), it could not be decided whether the ligand is carbonate or nitrate. However, a recent QM/MM study showed that it is most likely carbonate (Benediktsson & Bjornsson, 2020 ▸) and we came to the same conclusion with quantum refinement (Bergmann *et al.*, 2021 ▸). Here, we compare three different interpretations of the bidentate ligand, namely CO_3_^2−^, HCO_3_^−^ or NO_3_^−^. As in the previous section, we compare the results obtained using either the small QM model with only the directly coordinated groups or a larger model in which three neutralizing groups and one hydrogen-bonding group are added to the QM system, as is shown in Fig. 4 ▸. We also test three different values of the dielectric constant, namely 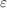 = 1, 4 and 80.

**Figure 5:**
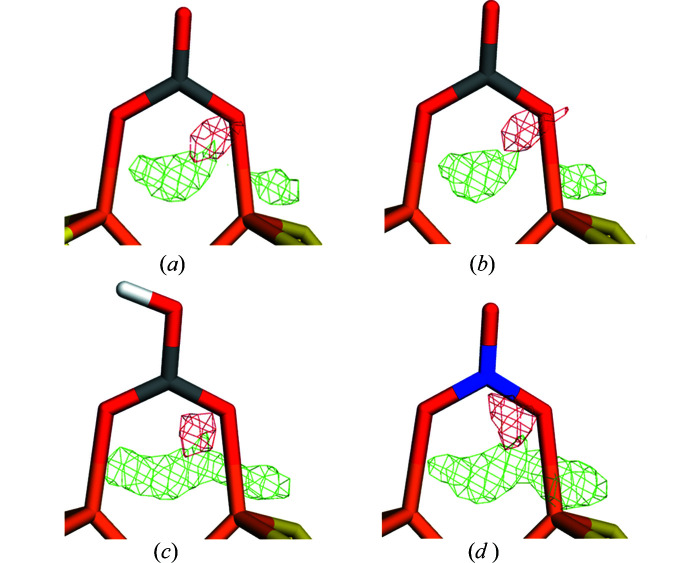
Quantum-refined structures of the bidentate ligand in the FeV cluster of V nitrogenase (small QM model) with different interpretations of the ligand: (*a*) CO_3_^2−^ in a vacuum, (*b*) CO_3_^2−^ with 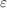 = 80, (*c*) HCO_3_^−^ with 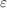 = 80 and (*d*) NO_3_^−^ with 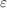 = 80. The *mF*_o_ − *DF*_c_ difference maps are contoured at +3σ (green) and −3σ (red).

The results are collected in Table 2 ▸. It can be seen that the RSZD score of the bidentate ligand (‘Lig’ in Table 2 ▸) is always lower for CO_3_^2−^ than for the other two ligands with both system sizes and all values of 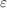 (by 0.7–2.8 for the small QM system and 0.2–0.7 for the large QM system). The same applies also for the sum of the RSZD scores (it is lower by 0.4–3.2; ‘Sum’ in Table 2 ▸). This can also be seen from the electron-density difference maps in Fig. 5 ▸, showing larger volumes of positive density (green) around the bidentate ligand for HCO_3_^−^ [part (*c*)] and NO_3_^−^ [part (*d*)] than for CO_3_^2−^ [parts (*a*) and (*b*)]. Thus, the COSMO model does not affect the discriminatory power of the quantum refinements and it does not change the conclusion that the bidentate model is CO_3_^2−^.

However, the strain energies become appreciably smaller for the COSMO calculations, especially for the small QM system (it decreases by 53–73 kJ mol^−1^ between a vacuum and 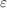 = 4. They therefore also become more similar for the three different interpretations of the bidentate ligand. As an effect, the strain energy becomes slightly smaller for the HCO_3_^−^ ligand than for CO_3_^2−^ for the small QM system (by less than 1 kJ mol^−1^) and slightly smaller for the NO_3_^−^ ligand than for CO_3_^2−^ for the large QM system (by 2–3 kJ mol^−1^). This is partly an effect of the larger negative charge of the CO_3_^2−^ ligand, which increases the strain energy (Ryde, 2002 ▸).

Moreover, it can be seen that in most cases, the sum of the RSZD scores of the active site decreases when the continuum-solvent model is added. The effect is clearest for the small QM system, whereas for the large QM system, the results are less smooth. The individual residues show more varying trends, but for the bidentate ligand itself, the results improve systematically as 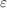 is increased for both the large and small QM systems with the preferred CO_3_^2−^ ligand, but not always for the incorrect ligands.

However, the most important question is whether the small QM systems with a COSMO model gives better results than the large system without any COSMO model. The results in Table 2 ▸ show that this is the case for the CO_3_^2−^ and HCO_3_^−^ ligands, whereas for NO_3_^−^, the sum of the RSZD scores is better only for 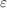 = 80, which is acceptable, because this is not the correct ligand. Thus, we conclude that also for this system is it favourable to employ an implicit solvent model in the quantum-refinement calculations.

### 3.3. Protonation of homocitrate in Mo nitrogenase

**Figure 6:**
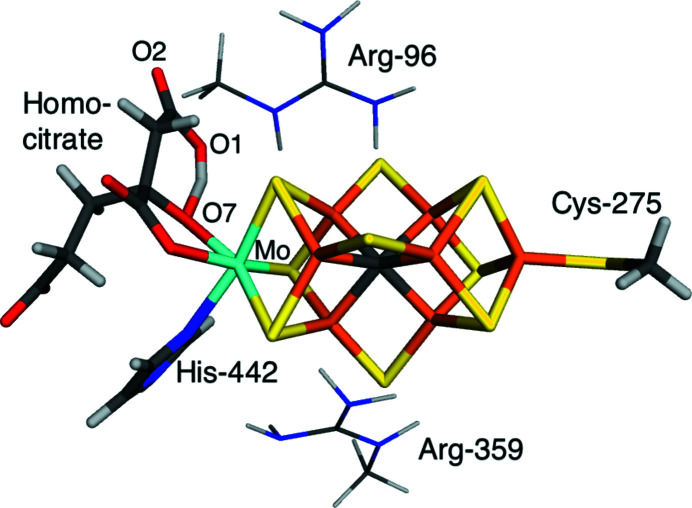
The QM systems used for the FeMo cluster. The small QM model contained only atoms shown with thick sticks, whereas the large QM model included also the two residues shown in thin sticks.

Next, we studied the protonation state of homocitrate in Mo nitrogenase, based on the 3u7q crystal structure (1.0 Å resolution). Homocitrate is a bidentate ligand of the Mo ion in the catalytic FeMo cluster (Fig. 6 ▸). It contains one alcohol and three carboxylate groups. Previous QM/MM, QM and quantum-refinement studies have suggested that in the resting E_0_ state, there is a proton on the alcohol group (O7 in Fig. 6 ▸), directed towards and almost shared with an O atom of one of the carboxylate groups (O1) (Benediktsson & Bjornsson, 2017 ▸; Cao *et al.*, 2017 ▸). However, a structure with an additional proton on the other O atom of that carboxylate group (O2) is competitive (Cao *et al.*, 2017 ▸). In this study, we have studied these two states (denoted 1Ha and 2H, respectively) with quantum refinement. As for V nitrogenase, we used either a minimal QM system with only the FeMo cluster, or a larger QM system extended by two nearby Arg residues (*cf*. Fig. 6 ▸). The net charge was −5 or −4 for the small QM systems and −3 or −2 for the large QM systems. For all states, we performed calculations in a vacuum or in the COSMO continuum solvent with a dielectric constant of 4 or 80.

The results are collected in Table 3 ▸. It can be seen that the behaviour is similar to that for the FeV cluster. The strain energies in general decrease with increasing 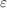 (with a single exception). The strain energies are always larger for the large QM systems. They are also normally larger for the 1Ha state than the 2H state, which reflects the larger negative charge of the 1Ha state.

Likewise, many of the RSZD scores decrease slightly with increasing 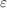 , but the variation is larger and varies with the considered group. For the homocitrate ligand, RSZD is lowest for 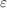 = 80 for all systems, except for the 1Ha state with the small QM system. The sum of the RSZD scores of the four moieties of the active site is lowest for 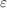 = 80 for two of the systems and for 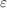 = 4 for the other two.

**Figure 7:**
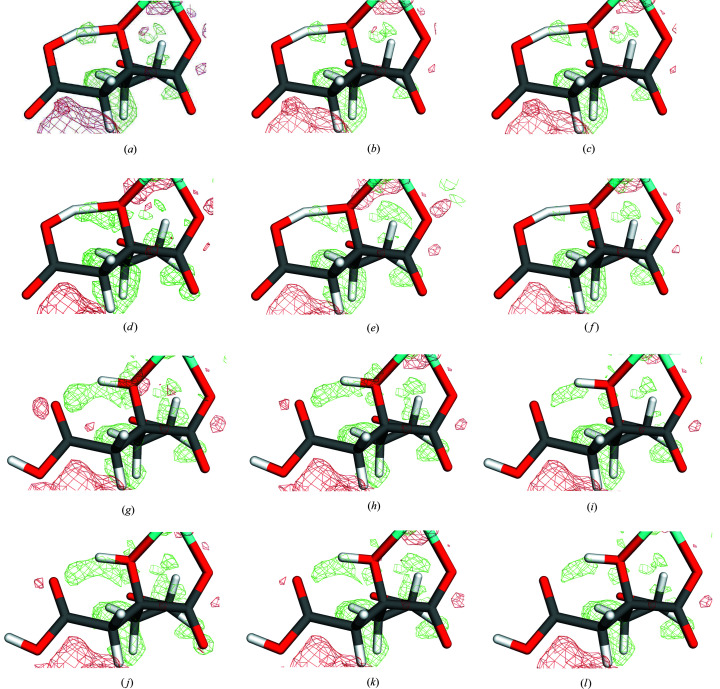
Quantum-refined structures of homocitrate in Mo nitrogenase: 1Ha with the small QM system in a vacuum (*a*), ɛ = 4 (*b*) and ɛ = 80 (*c*); 1Ha with the large QM system in a vacuum (*d*), ɛ = 4 (*e*) and ɛ = 80 (*f*); 2H with the small QM system in a vacuum (*g*), ɛ = 4 (*h*) and ɛ = 80 (*i*); 2H with the large QM system in a vacuum (*j*), ɛ = 4 (*k*) and ɛ = 80 (*l*). The *mF*_o_ − *DF*_c_ difference maps are contoured at +2.5σ (green) and −2.5σ (red).

However, the discrimination between the two protonation states is less clear. Our original study (Cao *et al.*, 2017 ▸) was based on the small model in a vacuum, for which 1Ha gives a slightly lower RSZD value for homocitrate (3.1 compared to 3.4). The results in Table 3 ▸, show that this is not the case for any of the other calculations. Still, the preference of 1Ha was mainly based on the electron-density difference maps, which show a positive density between the O1 and O7 atoms, and a negative density on the other side of O1 [Fig. 7 ▸ *(g*)], indicating that the increase in the O1–O7 distance caused by the protonation on O2 (from 2.47 to 2.60 Å; also listed in Table 3 ▸) is not supported by the crystallographic data. Figs. 7 ▸(*a*)–7(*f*) show that the positive density exists also for the 1Ha system, but at a lower level and with a smaller volume. The negative density is present only for 2H, but it is smaller for the large system and it decreases with 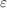 , reflecting that the O1–O7 distance decreases to 2.55 Å. Still, the difference maps around atoms O1, O2 and O7 are slightly better for 1Ha than for 2H even with the large system and 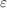 = 80. Therefore, we conclude that also for Mo nitrogenase, the results are improved by a continuum solvent, whereas the discriminatory power is somewhat decreased, but it was rather weak also without the continuum solvent.

### 3.4. pMMO

Our fourth test case is the Cu_B_ site of particulate (*i.e.* membrane-bound) methane monooxygenase (pMMO). The nature of the active site of this enzyme has been much debated (Ross & Rosenzweig, 2017 ▸). Based on low-resolution (≥2.6 Å) crystal structures, the Cu_B_ site was suggested to be the active site and it was modelled as a binuclear site, but with a distorted and strange geometry (Smith *et al.*, 2011 ▸). However, based on quantum refinement, we suggested that this site is mononuclear rather than binuclear (Cao *et al.*, 2018*a* ▸). More recently, it has been suggested that the active site is rather the Cu_C_ site, another mononuclear Cu site (Ross *et al.*, 2019 ▸; Peng *et al.*, 2021 ▸).

**Figure 8:**
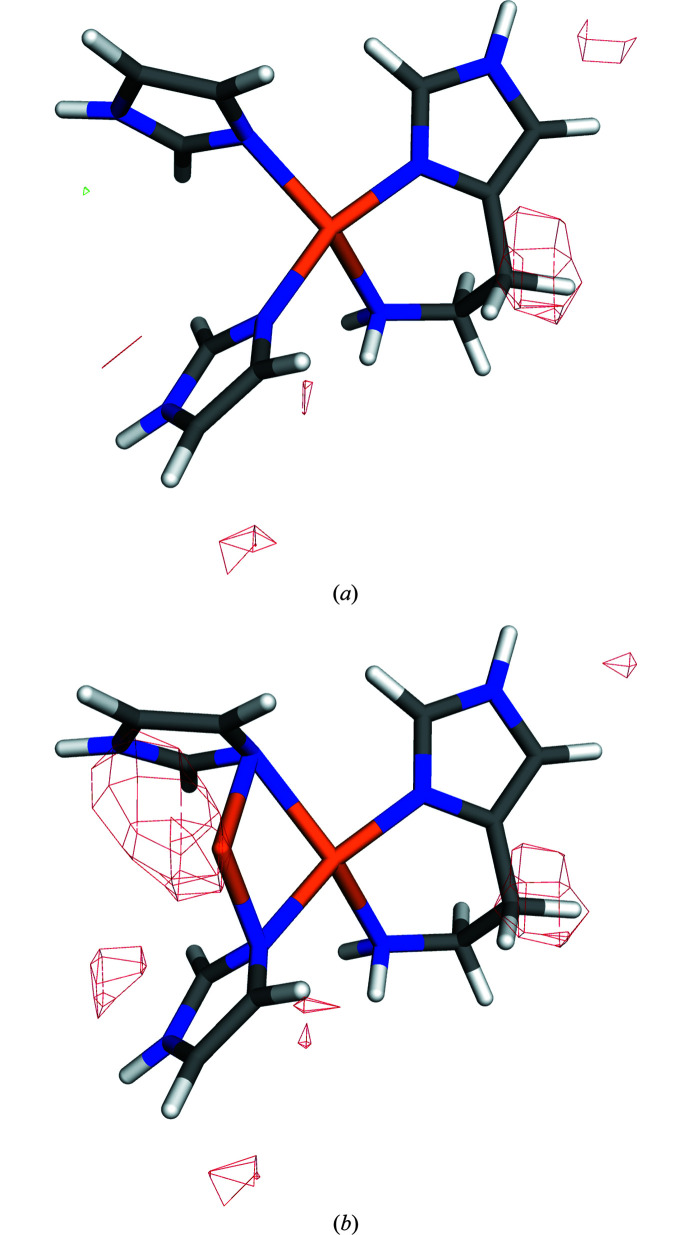
Quantum-refined structures of the Cu_B_ site in pMMO with (*a*) one or (*b*) two Cu ions. The *mF*_o_ − *DF*_c_ difference maps are contoured at +3σ (green) and −3σ (red).

In this study, we have refined the Cu_B_ site in the 2.8 Å 3rgb structure (Smith *et al.*, 2011 ▸) in a vacuum and with COSMO using 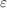 = 4 or 80. This was done for both a mononuclear and a binuclear site. Both sites were studied in the reduced, Cu^I^, state, giving a net charge of +1 for the mononuclear site and +2 for the binuclear site. The results are collected in Table 4 ▸. It can be seen that continuum solvent has a small effect on the RSZD scores of the three His ligands and the Cu ion(s), 0.1–0.4, without any clear trends. On the other hand, the strain energies decrease with increasing 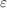 , somewhat more for the binuclear model than for the mononuclear model. Still, it is clear for all values of 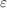 that the mononuclear model gives lower RSZD scores and lower strain energies than the binuclear model, in agreement with our original study (Cao *et al.*, 2018*a* ▸). This is also reflected by the electron-density difference maps in Fig. 8 ▸, showing extensive negative features around the extra Cu ion.

Thus, we conclude that for this structure at 2.8 Å resolution with a rather small charge of the QM system, there is no gain of using the continuum solvent, at least not for the RSZD values (but the results are not deteriorated either and strain energies decrease with increasing 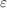 ).

### 3.5. Protonation states in acetylcholine esterase

**Figure 9:**
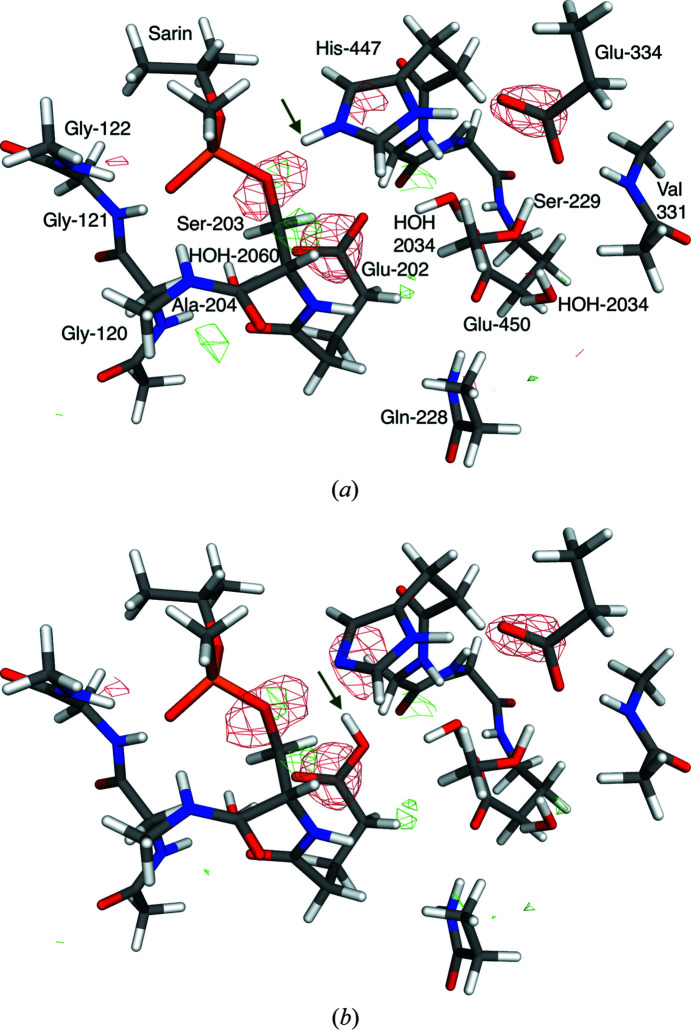
Quantum-refined structures of acetylcholine esterase phosphonylated by sarin in the (*a*) P0 and (*b*) P3 protonation states, using 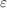 = 80. The *mF*_o_ − *DF*_c_ difference maps are contoured at +3σ (green) and −3σ (red). The green arrows indicate the position of the moved proton.

Finally, we tested the influence of the COSMO model on a system without metal ions for a study of the protonation state of the acetylcholine esterase active site. We used only one size of the QM system, involving 17 residues and three water molecules around the active-site Ser residue (Ser-203), which is modified by the covalent attachment of the nerve agent sarin (the net charge of the QM system is –2 e). We compared two different protonation states. In the first (P0), His-447 in the catalytic triad is doubly protonated (and therefore positively charged), whereas the two nearby residues Glu-202 and Glu-450 are deprotonated and negatively charged. In the second (P3; the numbering is taken from a study involving more states), the HE2 proton on His-447 is moved to Glu-202. The two states are shown in Fig. 9 ▸. In our original study (unpublished), we decided that P3 is the most likely state, although all quality measures did not point in the same direction. P0 was one of the competitive alternatives.

As for the other cases, we have run quantum refinement with three different values of the dielectric constant, namely 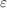 = 1, 4 and 80. The results are presented in Table 5 ▸. It can be seen that the RSZD values of the individual residues vary little with increasing 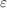 (up to 0.3, except for Ser-203 in the P0 state). Still, the small differences add up so that the sum of the RSZD scores is actually lowest for 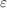 = 1 for both protonation states by 0.4–1.0. On the other hand, the strain energies mainly decrease with increasing 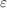 (except for P0 with 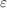 = 4).

Finally, we note that both the RSZD score and the strain energy point to P3 as the better protonation state, except for the strain energy in a vacuum. Likewise, the QM energy of the refined structures also point to P3 as the more stable state by 7–14 kJ mol^−1^, except for 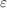 = 80, where P0 is 2 kJ mol^−1^ more stable. Thus, we can conclude that the continuum-solvation model has a relatively small effect on systems with the same net charge and differences in the protonation state of the organic molecules.

## 4. Conclusion

We have studied whether it is possible to improve the results of quantum refinement by performing the QM calculations in a continuum solvent. We have studied five crystal structures with different properties. For two different structures of V nitrogenase, we show that the continuum solvent strongly improves the results: both the RSZD scores and strain energies decrease when the continuum solvent is turned on. The best results are typically obtained with a high dielectric constant ( 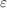 > 20). The improvement is largest with small QM systems. The reason for this is most likely the high negative charge of the small QM systems, namely −5 or −6. In particular, refinement of the small QM system in the continuum solvent gives results that are actually better than those obtained for the larger QM system in a vacuum, in which three positively charged residues have been added to reduce the large negative charge. We also show that the ability of the quantum-refinement calculations to discriminate between different interpretations of the structures is not affected by the continuum solvent. However, the difference in strain energies between different structural interpretations is typically reduced, because the strain energies become smaller in a continuum solvent.

For Mo nitrogenase, the results are similar: the strain energies still improve with increasing 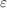 , and mostly also the RSZD scores. However, the difference between the two pro­ton­ation states (which was quite small already in a vacuum) becomes smaller.

For the other two crystal structures (particulate methane monooxygenase and acetylcholine esterase), the RSZD scores show little variation, but the strain energies in general decrease slightly with increasing 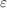 . Thus, we conclude that a continuum solvent typically is favourable only for structures with a large charge of the QM system. The continuum solvent can then be used to avoid the need of enlarging the QM system. However, for structures with a low resolution (>2 Å) or with a low charge of the QM system, there is no advantage of the continuum solvent.

## Supplementary Material

Coordinates of the QM systems of all structures discussed in the article. Zipped tar file. DOI: 10.1107/S2052520621009574/yj5002sup1.tgz

## Figures and Tables

**Table 1:** RSZD values and strain energies (Δ*E*_str_ in kJ mol^−1^) for the FeV cluster with different sizes of the QM system and different interpretations of the unknown ligand, replacing S2B (OH^−^ or NH^2−^), in quantum-refinement calculations without (\varepsilon = 1) or with a COSMO continuum solvent with two values of the dielectric constant (\varepsilon) The structure shows two alternative conformations, one with S2B intact (AC2, 14% occupancy) and one with it replaced by the unknown ligand (AC1, 86% occupancy). Gln-176 also rotates between the two conformations. ‘Sum’ is the sum of the RSZD scores for the six residues shown in the table. *Q* is the charge of the QM system.

				RSZD AC1			RSZD AC2				Δ*E*_str_	
Ligand	QM system	*Q*	\varepsilon	Gln-176	His-180	Lig	Gln-176	His-180	S2B	Sum RSZD	AC1	AC2
OH^−^	Small	−5	1	9.8	1.2	0.9	6.0	0.1	0.3	18.3	123.9	9.9
			4	6.3	1.1	0.0	5.6	0.0	0.4	13.4	43.7	8.9
			80	4.5	1.0	0.0	5.1	0.0	0.6	11.2	46.2	7.9
	Large	−2	1	5.6	1.0	0.5	4.7	0.0	0.9	12.7	208.1	20.4
			4	4.4	0.9	0.4	4.8	0.0	1.4	11.9	93.5	10.7
			80	3.8	1.0	0.4	4.7	0.0	1.2	11.1	64.1	10.1
NH^2−^	Small	−6	1	12.3	1.3	4.5	5.9	0.1	3.4	27.5	147.7	9.5
			4	9.5	1.2	1.3	5.8	0.0	0.7	18.5	51.7	7.5
			80	8.9	1.2	1.4	5.2	0.0	0.8	17.5	48.6	7.9
	Large	−3	1	8.0	1.1	4.0	4.6	0.0	4.8	22.5	222.4	20.7
			4	6.5	1.1	4.3	4.7	0.0	5.2	21.8	97.3	9.9
			80	5.8	1.0	3.8	4.7	0.0	5.4	20.7	71.3	10.0

**Table 2:** RSZD values and strain energies (Δ*E*_str_ in kJ mol^−1^) for the FeV cluster with different interpretations of the bidentate ligand and different sizes of the QM region (*cf*. Fig. 4 ▸) For all systems, calculations without (\varepsilon = 1) or with a COSMO continuum solvent with two values of the dielectric constant (\varepsilon) were tested. *Q* is the charge of the QM system. ‘Sum’ is the sum of the RSZD scores for the five residues shown in the table.

	QM syst	*Q*	\varepsilon	Cys-257	His-423	HCA	FeV	Lig	Sum	Δ*E*_str_
CO_3_^2−^	Small	−6	1	0.3	0.8	1.6	3.9	1.1	7.7	75.5
			4	0.4	0.9	1.5	3.2	0.9	6.9	23.0
			80	0.5	0.9	1.2	3.5	0.8	6.9	22.3
	Large	−3	1	0.6	0.8	1.4	3.0	1.5	7.3	95.0
			4	0.8	0.8	1.7	3.4	1.1	7.8	74.5
			80	0.8	0.7	1.8	2.6	0.8	6.7	61.6
HCO_3_^−^	Small	−5	1	0.3	0.9	1.6	4.7	2.2	9.7	95.4
			4	0.4	0.8	0.8	1.4	3.7	7.9	22.2
			80	0.5	0.8	1.1	3.4	1.5	7.3	21.6
	Large	−2	1	0.6	0.8	1.3	4.0	1.9	8.6	95.6
			4	0.7	0.8	1.7	3.7	1.6	8.5	78.1
			80	0.8	0.6	3.2	3.8	1.5	9.9	63.8
NO_3_^−^	Small	−5	1	0.6	0.8	1.3	4.1	2.1	8.9	90.3
			4	0.8	0.9	1.1	4.0	1.9	8.7	23.9
			80	0.8	0.9	1.1	4.0	1.9	8.2	25.0
	Large	−2	1	0.7	0.8	1.3	3.9	1.7	8.4	95.9
			4	0.7	0.8	1.6	3.4	1.8	8.3	71.6
			80	1.0	0.9	1.8	3.9	1.4	9.0	60.0

**Table 3:** RSZD values, O1–O7 distances (Å) and strain energies (Δ*E*_str_ in kJ mol^−1^) for the FeMo cluster with different protonation states of the homocitrate ligand (HCA) and different sizes of the QM region (small or large QM system, *cf*. Fig. 6 ▸) For all systems, calculations without (\varepsilon = 1) or with a COSMO continuum solvent with two values of the dielectric constant (\varepsilon) were tested. *Q* is the charge of the various QM systems. ‘Sum’ is the sum of the RSZD scores for the four groups shown in the table, whereas the RSZD scores of the homocitrate O1 and O7 atoms are also shown separately.

State	QM	*Q*	\varepsilon	Cys-275	His-442	HCA	FeMo	Sum	O1	O7	O1—O7	Δ*E*_str_
1Ha	Small	−5	1	0.7	2.1	3.1	6.7	12.6	0.3	0.8	2.47	109.3
			4	0.6	1.5	3.8	7.6	13.5	0.5	0.8	2.46	32.1
			80	0.7	1.1	3.6	6.7	12.1	0.9	1.3	2.46	34.2
	Large	−3	1	1.3	2.1	4.9	8.1	16.4	0.5	2.3	2.52	316.1
			4	1.0	1.6	3.6	7.2	13.4	0.5	2.4	2.49	120.5
			80	0.8	1.4	3.5	7.8	13.5	0.8	1.2	2.47	84.2
2H	Small	−4	1	0.9	1.6	3.4	6.0	11.9	3.3	2.3	2.60	75.0
			4	0.7	1.2	2.4	6.5	10.8	2.2	2.1	2.57	37.3
			80	0.9	1.1	2.3	6.6	10.9	1.4	1.4	2.55	32.2
	Large	−2	1	0.9	1.4	3.4	6.5	12.2	2.2	1.8	2.58	259.9
			4	0.7	1.2	2.4	7.0	11.3	1.9	1.8	2.56	111.9
			80	0.9	1.0	2.3	6.4	10.6	1.3	1.3	2.55	80.9

**Table 4:** RSZD values and strain energies (Δ*E*_str_ in kJ mol^−1^) for the Cu_B_ site in pMMO with one or two Cu ions For both models, calculations without (\varepsilon = 1) or with a COSMO continuum solvent with two values of the dielectric constant (\varepsilon) were tested. ‘Sum’ is the sum of the RSZD scores for the four residues shown in the table. *Q* is the net charge of the QM system.

#Cu	*Q*	\varepsilon	His-33	His-137	His-139	Cu	Sum	Δ*E*_str_
1	+1	1	1.2	1.1	1.2	1.1	4.6	6.4
		4	1.1	0.8	1.4	0.9	4.2	5.5
		80	1.5	1.1	1.4	1.3	5.3	4.2
2	+2	1	1.4	1.1	1.7	3.6	7.8	81.7
		4	1.1	1.0	1.7	3.5	7.3	77.0
		80	1.2	1.0	1.5	3.8	7.5	74.0

**Table 5:** RSZD values for all residues involved in the QM system for acetylcholine esterase, as well as the strain energies (Δ*E*_str_) and the difference in QM energy of the P0 and P3 protonation states (Δ*E*_QM1_, both in kJ mol^−1^) ‘Sum’ is the sum of the RSZD scores of all the residues shown in the table.

State	P0			P3		
	ɛ = 1	ɛ = 4	ɛ = 80	ɛ = 1	ɛ = 4	ɛ = 80
Tyr-119	1.5	1.5	1.5	1.6	1.6	1.5
Gly-120	1.3	1.6	1.6	1.3	1.4	1.5
Gly-121	0.1	0.2	0.2	0.1	0.2	0.2
Gly-122	1.2	1.2	1.3	1.1	1.2	1.2
Glu-202	0.5	0.3	0.4	0.5	0.5	0.5
Ser-203	4.2	3.7	3.6	4.1	4.1	4.0
Ala-204	1.1	1.2	1.2	1.0	1.0	1.0
Gln-228	2.1	2.1	2.1	2.1	2.1	2.1
Ser-229	1.3	1.4	1.5	1.3	1.3	1.4
Val-330	0.9	1.1	1.2	0.9	1.1	1.2
Val-331	0.0	0.0	0.0	0.0	0.0	0.0
Glu-334	1.4	1.4	1.4	1.4	1.4	1.5
His-447	0.4	0.6	0.6	0.6	0.7	0.7
Gly-448	1.5	1.3	1.4	1.3	1.4	1.5
Tyr-449	2.2	2.1	2.0	2.2	2.2	2.1
Glu-450	2.1	2.0	1.9	2.1	2.1	2.1
HOH-2032	1.0	0.9	0.9	0.5	0.4	0.4
HOH-2034	1.7	1.9	2.0	1.7	1.9	1.9
HOH-2060	2.8	2.9	2.9	2.8	2.7	2.8
Sum	27.3	27.4	27.7	26.6	27.3	27.6
Δ*E*_str_	44.5	52.1	39.7	47.1	37.7	34.8
Δ*E*_QM1_	7.3	14.4	−2.4	0.0	0.0	0.0

## References

[bb1] Adams, P. D., Pannu, N. S., Read, R. J. & Brünger, A. T. (1997). *Proc. Natl Acad. Sci.* **94**, 5018–5023. 10.1073/pnas.94.10.5018 PMC24623 9144182

[bb2] Afonine, P. V., Grosse-Kunstleve, R. W., Echols, N., Headd, J. J., Moriarty, N. W., Mustyakimov, M., Terwilliger, T. C., Urzhumtsev, A., Zwart, P. H. & Adams, P. D. (2012). *Acta Cryst.* D**68**, 352–367. 10.1107/S0907444912001308 PMC3322595 22505256

[bb3] Allgardsson, A., Berg, L., Akfur, C., Hörnberg, A., Worek, F., Linusson, A. & Ekström, F. J. (2016). *Proc. Natl Acad. Sci. USA*, **113**, 5514–5519. 10.1073/pnas.1523362113 PMC4878515 27140636

[bb4] Benediktsson, B. & Bjornsson, R. (2017). *Inorg. Chem.* **56**, 13417–13429. 10.1021/acs.inorgchem.7b02158 29053260

[bb5] Benediktsson, B. & Bjornsson, R. (2020). *Inorg. Chem.* **59**, 11514–11527. 10.1021/acs.inorgchem.0c01320 PMC7458435 32799489

[bb6] Benediktsson, B., Thorhallsson, A. T. & Bjornsson, R. (2018). *Chem. Commun.* **54**, 7310–7313. 10.1039/c8cc03793k 29882938

[bb7] Bergmann, J., Davidson, M., Oksanen, E., Ryde, U. & Jayatilaka, D. (2020). *IUCrJ*, **7**, 158–165. 10.1107/S2052252519015975 PMC7055371 32148844

[bb8] Bergmann, J., Oksanen, E. & Ryde, U. (2021). *J. Inorg. Biochem.* **219**, 111426. 10.1016/j.jinorgbio.2021.111426 33756394

[bb9] Bjornsson, R., Lima, F. A., Spatzal, T., Weyhermüller, T., Glatzel, P., Bill, E., Einsle, O., Neese, F. & DeBeer, S. (2014). *Chem. Sci.* **5**, 3096–3103.

[bb10] Bjornsson, R., Neese, F. & DeBeer, S. (2017). *Inorg. Chem.* **56**, 1470–1477. 10.1021/acs.inorgchem.6b02540 28071903

[bb11] Borbulevych, O., Martin, R. I., Tickle, I. J. & Westerhoff, L. M. (2016). *Acta Cryst.* D**72**, 586–598. 10.1107/S2059798316002837 PMC4822566 27050137

[bb12] Borbulevych, O. Y., Martin, R. I. & Westerhoff, L. M. (2021). *J. Comput. Aided Mol. Des.* **35**, 433–451. 10.1007/s10822-020-00354-6 PMC8018927 33108589

[bb13] Borbulevych, O. Y., Plumley, J. A., Martin, R. I., Merz, K. M. & Westerhoff, L. M. (2014). *Acta Cryst.* D**70**, 1233–1247. 10.1107/S1399004714002260 PMC4014119 24816093

[bb14] Brunger, A. T. (2007). *Nat. Protoc.* **2**, 2728–2733. 10.1038/nprot.2007.406 18007608

[bb15] Brünger, A. T., Adams, P. D., Clore, G. M., DeLano, W. L., Gros, P., Grosse-Kunstleve, R. W., Jiang, J.-S., Kuszewski, J., Nilges, M., Pannu, N. S., Read, R. J., Rice, L. M., Simonson, T. & Warren, G. L. (1998). *Acta Cryst.* D**54**, 905–921. 10.1107/s0907444998003254 9757107

[bb16] Caldararu, O., Feldt, M., Cioloboc, D., van Severen, M.-C., Starke, K., Mata, R. A., Nordlander, E. & Ryde, U. (2018). *Sci. Rep.* **8**, 4684. 10.1038/s41598-018-22751-6 PMC5856855 29549261

[bb17] Cao, L., Börner, M. C., Bergmann, J., Caldararu, O. & Ryde, U. (2019). *Inorg. Chem.* **58**, 9672–9690. 10.1021/acs.inorgchem.9b00400 31282663

[bb18] Cao, L., Caldararu, O., Rosenzweig, A. C. & Ryde, U. (2018*a*). *Angew. Chem. Int. Ed.* **57**, 162–166. 10.1002/anie.201708977 PMC5808928 29164769

[bb19] Cao, L., Caldararu, O. & Ryde, U. (2017). *J. Phys. Chem. B*, **121**, 8242–8262. 10.1021/acs.jpcb.7b02714 28783353

[bb20] Cao, L., Caldararu, O. & Ryde, U. (2018*b*). *J. Chem. Theory Comput.* **14**, 6653–6678. 10.1021/acs.jctc.8b00778 30354152

[bb21] Cao, L., Caldararu, O. & Ryde, U. (2020). *J. Biol. Inorg. Chem.* **25**, 847–861. 10.1007/s00775-020-01813-z PMC7511287 32856107

[bb22] Cao, L. & Ryde, U. (2018). *Int. J. Quantum Chem.* **118**, e25627.

[bb23] Cao, L. & Ryde, U. (2020). *Acta Cryst.* D**76**, 1145–1156. 10.1107/S2059798320012917 PMC7604908 33135685

[bb24] Capelli, S. C., Bürgi, H.-B., Dittrich, B., Grabowsky, S. & Jayatilaka, D. (2014). *IUCrJ*, **1**, 361–379. 10.1107/S2052252514014845 PMC4174878 25295177

[bb25] Cromer, D. T. (1965). *Acta Cryst.* **19**, 224–227.

[bb26] Dittrich, B., Hübschle, C. B., Pröpper, K., Dietrich, F., Stolper, T. & Holstein, J. J. (2013). *Acta Cryst.* B**69**, 91–104. 10.1107/S2052519213002285 23719696

[bb27] Domagała, S., Fournier, B., Liebschner, D., Guillot, B. & Jelsch, C. (2012). *Acta Cryst.* A**68**, 337–351. 10.1107/S0108767312008197 22514066

[bb28] Eichkorn, K., Treutler, O., Öhm, H., Häser, M. & Ahlrichs, R. (1995). *Chem. Phys. Lett.* **240**, 283–290.

[bb29] Eichkorn, K., Weigend, F., Treutler, O. & Ahlrichs, R. (1997). *Theoretical Chemistry Accounts: Theory, Computation, and Modeling (Theor. Chim. Acta)*, **97**, 119–124.

[bb30] Engh, R. A. & Huber, R. (1991). *Acta Cryst.* A**47**, 392–400.

[bb31] Fadel, F., Zhao, Y., Cachau, R., Cousido-Siah, A., Ruiz, F. X., Harlos, K., Howard, E., Mitschler, A. & Podjarny, A. (2015). *Acta Cryst.* D**71**, 1455–1470. 10.1107/S139900471500783X 26143917

[bb32] Furche, F., Ahlrichs, R., Hättig, C., Klopper, W., Sierka, M. & Weigend, F. (2014). *WIREs Comput. Mol. Sci.* **4**, 91–100.

[bb33] Genoni, A., Bučinský, L., Claiser, N., Contreras–García, J., Dittrich, B., Dominiak, P. M., Espinosa, E., Gatti, C., Giannozzi, P., Gillet, J.-M., Jayatilaka, D., Macchi, P., Madsen, A., Massa, L., Matta, C. F., Merz, K. M. Jr, Nakashima, P. N. H., Ott, H., Ryde, U., Schwarz, K., Sierka, M. & Grabowsky, S. (2018). *Chem. Eur. J.* **24**, 10881–10905. 10.1002/chem.201705952 29488652

[bb34] Greco, C., Fantucci, P., Ryde, U. & de Gioia, L. (2011). *Int. J. Quant. Chem.* **111**, 3949–3960.

[bb35] Grimme, S., Antony, J., Ehrlich, S. & Krieg, H. (2010). *J. Chem. Phys.* **132**, 154104. 10.1063/1.3382344 20423165

[bb36] Grimme, S., Ehrlich, S. & Goerigk, L. (2011). *J. Comput. Chem.* **32**, 1456–1465. 10.1002/jcc.21759 21370243

[bb37] Hansen, N. K. & Coppens, P. (1978). *Acta Cryst.* A**34**, 909–921.

[bb38] Hoffman, B. M., Lukoyanov, D., Yang, Z.-Y., Dean, D. R. & Seefeldt, L. C. (2014). *Chem. Rev.* **114**, 4041–4062. 10.1021/cr400641x PMC4012840 24467365

[bb39] Hsiao, Y.-W., Sanchez-Garcia, E., Doerr, M. & Thiel, W. (2010). *J. Phys. Chem. B*, **114**, 15413–15423. 10.1021/jp108095n 20977248

[bb40] Hu, L. & Ryde, U. (2011). *J. Chem. Theory Comput.* **7**, 2452–2463. 10.1021/ct100725a 26606619

[bb41] Jarzembska, K. N. & Dominiak, P. M. (2012). *Acta Cryst.* A**68**, 139–147. 10.1107/S0108767311042176 22186290

[bb42] Jayatilaka, D. & Dittrich, B. (2008). *Acta Cryst.* A**64**, 383–393. 10.1107/S0108767308005709 18421128

[bb43] Klamt, A., Jonas, V., Bürger, T. & Lohrenz, J. C. (1998). *J. Phys. Chem. A*, **102**, 5074–5085.

[bb44] Klamt, A. & Schüürmann, G. (1993). *J. Chem. Soc. Perkin Trans. 2*, pp. 799–805.

[bb45] Kleywegt, G. J. (2007). *Acta Cryst.* D**63**, 94–100. 10.1107/S0907444906022657 PMC2483469 17164531

[bb46] Kleywegt, G. J. & Jones, T. A. (1997). *Methods Enzymol.* **227**, 208–230. 10.1016/s0076-6879(97)77013-7 18488311

[bb47] Lovell, T., Li, J., Liu, T., Case, D. A. & Noodleman, L. (2001). *J. Am. Chem. Soc.* **123**, 12392–12410. 10.1021/ja011860y 11734043

[bb48] Malaspina, L. A., Wieduwilt, E. K., Bergmann, J., Kleemiss, F., Meyer, B., Ruiz-López, M. F., Pal, R., Hupf, E., Beckmann, J., Piltz, R. O., Edwards, A. J., Grabowsky, S. & Genoni, A. (2019). *J. Phys. Chem. Lett.* **10**, 6973–6982. 10.1021/acs.jpclett.9b02646 31633355

[bb49] Nilsson, K. & Ryde, U. (2004). *J. Inorg. Biochem.* **98**, 1539–1546. 10.1016/j.jinorgbio.2004.06.006 15337606

[bb50] Pannu, N. S. & Read, R. J. (1996). *Acta Cryst.* A**52**, 659–668.

[bb51] Peng, W., Qu, X., Shaik, S. & Wang, B. (2021). *Nat. Catal.* **4**, 266–273.

[bb52] Ross, M. O., MacMillan, F., Wang, J., Nisthal, A., Lawton, T. J., Olafson, B. D., Mayo, S. L., Rosenzweig, A. C. & Hoffman, B. M. (2019). *Science*, **364**, 566–570. 10.1126/science.aav2572 PMC6664434 31073062

[bb53] Ross, M. O. & Rosenzweig, A. C. (2017). *J. Biol. Inorg. Chem.* **22**, 307–319. 10.1007/s00775-016-1419-y PMC5352483 27878395

[bb54] Rulíšek, L. & Ryde, U. (2006). *J. Phys. Chem. B*, **110**, 11511–11518. 10.1021/jp057295t 16771427

[bb55] Ryde, U. (2002). In *Recent Research Developments in Protein Engineering*, Vol. 2, pp. 65–91. Trivandrum: Research Signpost.

[bb56] Ryde, U. (2016). *Methods Enzymol.* **577**, 119–158. 10.1016/bs.mie.2016.05.014 27498637

[bb57] Ryde, U. & Nilsson, K. (2003). *J. Am. Chem. Soc.* **125**, 14232–14233. 10.1021/ja0365328 14624544

[bb58] Ryde, U., Olsen, L. & Nilsson, K. (2002). *J. Comput. Chem.* **23**, 1058–1070. 10.1002/jcc.10093 12116392

[bb59] Senn, H. M. & Thiel, W. (2009). *Angew. Chem. Int. Ed.* **48**, 1198–1229. 10.1002/anie.200802019 19173328

[bb60] Sigfridsson, E. & Ryde, U. (1998). *J. Comput. Chem.* **19**, 377–395.

[bb61] Sippel, D. & Einsle, O. (2017). *Nat. Chem. Biol.* **13**, 956–960. 10.1038/nchembio.2428 PMC5563456 28692069

[bb62] Sippel, D., Rohde, M., Netzer, J., Trncik, C., Gies, J., Grunau, K., Djurdjevic, I., Decamps, L., Andrade, S. L. A. & Einsle, O. (2018). *Science*, **359**, 1484–1489. 10.1126/science.aar2765 29599235

[bb63] Smith, S. M., Rawat, S., Telser, J., Homan, B. M., Stemmler, T. L. & Rosenzweig, A. C. (2011). *Biochemistry*, **50**, 10231–10240. 10.1021/bi200801z PMC3364217 22013879

[bb64] Söderhjelm, P. & Ryde, U. (2006). *J. Mol. Struct. Theochem*, **770**, 199–219.

[bb65] Spatzal, T., Aksoyoglu, M., Zhang, L., Andrade, S. L. A., Schleicher, E., Weber, S., Rees, D. C. & Einsle, O. (2011). *Science*, **334**, 940. 10.1126/science.1214025 PMC3268367 22096190

[bb66] Szilagyi, R. K. & Winslow, M. A. (2006). *J. Comput. Chem.* **27**, 1385–1397. 10.1002/jcc.20449 16788911

[bb67] Tao, J., Perdew, J. P., Staroverov, V. N. & Scuseria, G. E. (2003). *Phys. Rev. Lett.* **91**, 146401. 10.1103/PhysRevLett.91.146401 14611541

[bb68] Tickle, I. J. (2012). *Acta Cryst.* D**68**, 454–467. 10.1107/S0907444911035918 PMC3322605 22505266

[bb69] Weigend, F. & Ahlrichs, R. (2005). *Phys. Chem. Chem. Phys.* **7**, 3297–3305. 10.1039/b508541a 16240044

[bb70] Winn, M. D., Ballard, C. C., Cowtan, K. D., Dodson, E. J., Emsley, P., Evans, P. R., Keegan, R. M., Krissinel, E. B., Leslie, A. G. W., McCoy, A., McNicholas, S. J., Murshudov, G. N., Pannu, N. S., Potterton, E. A., Powell, H. R., Read, R. J., Vagin, A. & Wilson, K. S. (2011). *Acta Cryst.* D**67**, 235–242. 10.1107/S0907444910045749 PMC3069738 21460441

[bb71] Yu, N., Hayik, S. A., Wang, B., Liao, N., Reynolds, C. H. & Merz, K. M. (2006). *J. Chem. Theory Comput.* **2**, 1057–1069. 10.1021/ct0600060 PMC2600719 19079786

[bb72] Yu, N., Yennawar, H. P. & Merz, K. M. (2005). *Acta Cryst.* D**61**, 322–332. 10.1107/S0907444904033669 15735343

[bb73] Zheng, M., Reimers, J. R., Waller, M. P. & Afonine, P. V. (2017). *Acta Cryst.* D**73**, 45–52. 10.1107/S2059798316019847 PMC5331472 28045384

